# Risk stratification and prognostic performance of the predisposition, infection, response, and organ dysfunction (PIRO) scoring system in septic patients in the emergency department: a cohort study

**DOI:** 10.1186/cc13832

**Published:** 2014-04-16

**Authors:** Yun-Xia Chen, Chun-Sheng Li

**Affiliations:** 1 Emergency Department of Beijing Chao-Yang Hospital, Affiliated to Capital Medical University, Chaoyang District, Beijing 100020, China

## Abstract

**Introduction:**

The predisposition, infection, response and organ dysfunction (PIRO) staging system was designed as a stratification tool to deal with the inherent heterogeneity of septic patients. The present study was conducted to assess the performance of PIRO in predicting multiple organ dysfunction (MOD), intensive care unit (ICU) admission, and 28-day mortality in septic patients in the emergency department (ED), and to compare this scoring system with the Mortality in Emergency Department Sepsis (MEDS) and Acute Physiology and Chronic Health Evaluation (APACHE II) scores.

**Methods:**

Consecutive septic patients (*n* = 680) admitted to the ED of Beijing Chao-Yang Hospital were enrolled. PIRO, MEDS, and APACHE II scores were calculated for each patient on ED arrival. Organ function was reassessed within 3 days of enrollment. All patients were followed up for 28 days. Outcome criteria were the development of MOD within 3 days, ICU admission or death within 28 days after enrollment. The predictive ability of the four components of PIRO was analyzed separately. Receiver operating characteristic (ROC) curve and logistic regression analysis were used to assess the prognostic and risk stratification value of the scoring systems.

**Results:**

Organ dysfunction independently predicted ICU admission, MOD, and 28-day mortality, with areas under the ROC curve (AUC) of 0.888, 0.851, and 0.816, respectively. The predictive value of predisposition, infection, and response was weaker than that of organ dysfunction. A negative correlation was found between the response component and MOD, as well as mortality. PIRO, MEDS, and APACHE II scores significantly differed between patients who did and did not meet the outcome criteria (*P* < 0.001). PIRO and APACHE II independently predicted ICU admission and MOD, but MEDS did not. All three systems were independent predictors of 28-day mortality with similar AUC values. The AUC of PIRO was 0.889 for ICU admission, 0.817 for MOD, and 0.744 for 28-day mortality. The AUCs of PIRO were significantly greater than those of APACHE II and MEDS (*P* < 0.05) in predicting ICU admission and MOD.

**Conclusions:**

The study indicates that PIRO is helpful for risk stratification and prognostic determinations in septic patients in the ED.

## Introduction

Sepsis, which can progress to severe sepsis and septic shock, is becoming a major healthcare problem and affects millions of people around the world each year [1-5]. The emergency department (ED) is a common location for the initial evaluation and management of septic patients. However, evaluation is complicated by the heterogeneity of clinical manifestations, sites of infection, comorbid conditions, and etiologic microorganisms [6]. Given the complexity of sepsis, biomarkers and mathematical models offer potential guidance once they have been carefully validated [7,8].

The concept of the predisposition, infection, response, and organ dysfunction (PIRO) scoring system was recommended at the 2001 International Sepsis Definitions Conference to improve the traditional classification of sepsis [9]. The PIRO system is an ideal staging system that incorporates assessment of premorbid baseline susceptibility (predisposition), the specific disorder responsible for illness (infection), the response of the host to infection, and the resulting degree of organ dysfunction. The four components of the PIRO system cover multiple known independent factors that may influence the onset, development, and outcome of sepsis. The system was proposed not as a prognostic measure, but rather as a stratification tool to resolve the inherent heterogeneity of septic patients. The PIRO system was expected to be helpful for risk stratification and for enrollment criteria in clinical studies, and to differentiate patients who may benefit from certain types of therapeutic intervention.

The PIRO system is theoretically an ideal stratification tool, but it is difficult to translate into clinical practice. In 2008, the first clinical PIRO model was developed [10], followed by another three different PIRO systems over the next 5 years. The variables contained in these PIRO systems varied markedly, partly because of different enrollment populations. Three studies enrolled septic patients admitted to the ICU and another enrolled patients with suspected infection in the ED. The PIRO systems were based on ICU populations with ICU-specific variables, such as the location and length of stay prior to ICU admission, the reason for ICU admission, and the infective agents, so they were not very suitable for application in the ED [10-13]. The PIRO system devised by Howell and colleagues was developed in patients in the ED with suspected infection and was designed for bedside use at clinical presentation [12]. The variables of this PIRO system were easily obtained in the ED, so its applicability was superior to others. Since the original study mainly focused on the prognostic value of PIRO, the system’s risk stratification ability needs further evaluation.

This study was designed to assess the predictive performance of the PIRO system for ICU admission, development of multiple organ dysfunction (MOD), and 28-day mortality in septic patients in the ED, and to compare this scoring system with the Acute Physiology and Chronic Health Evaluation (APACHE) II [14] and Mortality in Emergency Department Sepsis (MEDS) scores [7].

## Materials and methods

### Patients

We conducted a prospective observational clinical study in the ED of Beijing Chao-Yang Hospital, a teaching hospital of Capital Medical University with approximately 250,000 ED visits per year. The study was approved by the Beijing Chao-Yang hospital ethics committee, and written informed consent was obtained from every patient.

The enrollment criteria were age ≥ 18 years and fulfillment of the sepsis criteria as defined by the International Sepsis Definitions Conference [9]. The exclusion criteria were as follows: age < 18 years, terminal stage of disease (malignant cancer with metastases, AIDS, end-stage renal or hepatic disease, chronic heart failure), and refusal to participate in the study by patients or their relatives.

### Data collection

Patients’ basic information, including age, gender, comorbidities, telephone number, and medical record number, was recorded at the time of enrollment. Vital signs, laboratory results, and imaging results obtained on ED arrival were documented, together with diagnoses. The PIRO [12], APACHE II [14], and MEDS [7] scores were calculated using data obtained on ED arrival. The criteria for the four PIRO domains are presented in Table 1.

**Table 1:** Criteria of the PIRO system

**Variable**	**0**	**1**	**2**	**3**	**4**
Predisposition					
Age (years)	<65	65 to 80	>80		
COPD		Yes			
Liver disease			Yes		
Nursing home resident			Yes		
Malignancy		Without metastases	With metastases		
Infection					
Skin/soft tissue infection	Yes				
Any other infection			Yes		
Pneumonia					Yes
Response					
Respiratory rate (bpm)				>20	
Bands		>5%			
Heart rate (bpm)			>120		
Organ dysfunction					
SBP (mmHg)	>90		70 to 90		<70
BUN (mmol/l)			>7.1		
Respiratory failure/hypoxemia				Yes	
Lactate (mmol/l)				>4.0	
Platelet count (×10^9^/l)			<150		

BUN, blood urea nitrogen; COPD, chronic obstructive pulmonary disease; PIRO, predisposition, infection, response, and organ dysfunction; SBP, systolic blood pressure.

### Outcome variables

All patients were followed up for 28 days through medical records or by telephone. Organ function was assessed at enrollment and was reassessed when deterioration occurred or on the third day of enrollment if the patient was relatively stable. ICU admission during follow-up, development of MOD within 3 days of enrollment, and 28-day mortality were considered as the outcome criteria. MOD was defined as fulfillment of two or more of the criteria of severe sepsis at any time within 3 days of enrollment, excluding organ dysfunction, which was induced by pre-existing disease.

### Statistical analysis

All data were analyzed by SPSS version 16.0 (SPSS Inc., Chicago, IL, USA). Normally distributed data were expressed as the mean ± standard deviation and were compared using the independent-samples *t* test. Data with skewed distribution were expressed as the median and quartiles and were analyzed by the Mann–Whitney *U* test. The chi-square test was used for the comparison of frequencies. Logistic regression analysis was used to determine independent predictors of outcomes. Receiver operating characteristic (ROC) curves were constructed and the area under the ROC curves (AUC) was determined to assess predictive value. All statistical tests were two-tailed, and *P* < 0.05 was considered statistically significant.

## Results

### Characteristics of the study cohort

Eight hundred and thirty-seven consecutive septic patients were screened from November 2011 to October 2012. We excluded 153 patients with non-infectious disease and four patients with incomplete data, and thus included 680 septic patients in the study. The characteristics of the study cohort are presented in Table 2.

**Table 2:** Characteristics of the study cohort

Number of patients	680
Male (%)	61.2
Age (years)	73 (60 to 79)
Infection site	
Pneumonia (*n*)	467
Intra-abdominal infection (*n*)	170
Pyelonephritis	21
Central nervous system infection (*n*)	18
Other infections (*n*)	4
APACHE II score	17.0 ± 7.7
MEDS score	11 (8 to 16)
PIRO score	11 (9 to 14)
28-day mortality (%)	26.2
ICU admission (%)	21.8
Incidence of MOD within 3 days of enrollment (%)	34.4

APACHE, Acute Physiology and Chronic Health Evaluation; MEDS, Mortality in Emergency Department Sepsis; MOD, multiple organ dysfunction; PIRO, predisposition, infection, response, and organ dysfunction.

The mean age was much higher in patients subsequently admitted to the ICU (76 (range, 67 to 78) years) versus those who were not (72 (59 to 79) years, *P* = 0.012), in patients who developed MOD (75 (66 to 81) years) versus those who did not (71 (51 to 78) years, *P* < 0.001), and in patients who died within 28 days (74 (65 to 81) years) versus survivors (73 (59 to 79) years, *P* = 0.012). The percentage of males did not differ between patients with different outcomes.

### Risk stratification and prognostic performance of the PIRO system

**Figure 1:**
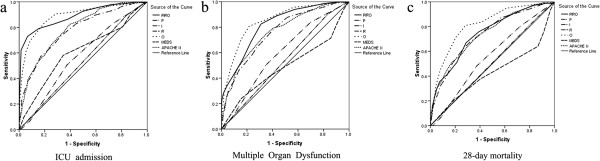
**Receiver operating characteristic curves of the PIRO, MEDS and APACHE II scores for predicting outcomes. (a)** ICU admission. **(b)** Development of multiple organ dysfunction. **(c)** Twenty-eight-day mortality. APACHE, Acute Physiology and Chronic Health Evaluation; MEDS, Mortality in Emergency Department Sepsis; PIRO, predisposition (P), infection (I), response (R), and organ dysfunction (O).

The predictive ability of each of the four components of the PIRO system was analyzed separately. The results are presented in Table 3. Predisposition, infection, response, and organ dysfunction were all independent predictors of ICU admission. Predisposition, infection, and organ dysfunction independently predicted MOD, but response did not. For 28-day mortality, predisposition, response, and organ dysfunction were the independent predictors, but infection was not. The ROC curves are shown in Figure 1, and the AUCs are presented in Table 4. The predictive ability of organ dysfunction was the best among the four components. The AUCs of organ dysfunction in predicting ICU admission, MOD, and 28-day mortality were 0.888, 0.851, and 0.816, respectively. A negative correlation was found between response and MOD, as well as between response and 28-day mortality.

**Table 3:** Predictive ability of the four components of the PIRO system

**Outcome variable**	**Predictor**	***B***	**SE**	**Wald**	***P *** **value**	**OR**	**95% CI for OR**	
							**5%**	**95%**
ICU admission	Predisposition	0.387	0.140	7.591	0.006	1.472	1.118	1.939
	Infection	0.440	0.210	4.382	0.036	1.553	1.028	2.344
	Response	0.364	0.093	15.351	0.000	1.439	1.200	1.727
	Organ dysfunction	0.665	0.058	132.428	0.000	1.944	1.736	2.177
	Constant	−7.743	1.031	56.440	0.000	0.000		
MOD	Predisposition	0.409	0.110	13.872	0.000	1.506	1.214	1.867
	Infection	0.378	0.166	5.228	0.022	1.460	1.056	2.020
	Response	−0.125	0.073	2.907	0.088	0.883	0.764	1.019
	Organ dysfunction	0.544	0.044	154.852	0.000	1.723	1.582	1.878
	Constant	−4.272	0.727	34.553	0.000	0.014		
28-day mortality	Predisposition	0.225	0.112	4.060	0.044	1.253	1.006	1.560
	Infection	−0.017	0.144	0.014	0.904	0.983	0.741	1.303
	Response	−0.331	0.073	20.345	0.000	0.718	0.622	0.829
	Organ dysfunction	0.410	0.037	125.453	0.000	1.507	1.403	1.620
	Constant	−1.906	0.591	10.391	0.001	0.149		

CI, confidence interval; MOD, multiple organ dysfunction; OR, odds ratio; PIRO, predisposition, infection, response, and organ dysfunction; SE, standard error.

**Table 4:** Areas under the receiver operating characteristic curves of predictors

**Outcome variable**	**Predictor**	**AUC**	**SE**	***P *** **value**	**95% CI for AUC**	
					**5%**	**95%**
ICU admission	PIRO	0.889	0.017	0.000	0.855	0.923
	Predisposition	0.570	0.026	0.009	0.520	0.621
	Infection	0.528	0.026	0.299	0.477	0.579
	Response	0.606	0.028	0.000	0.551	0.660
	Organ dysfunction	0.888	0.017	0.000	0.856	0.921
	MEDS	0.774**	0.022	0.000	0.731	0.817
	APACHE II	0.789**	0.020	0.000	0.750	0.829
MOD	PIRO	0.817	0.017	0.000	0.785	0.849
	Predisposition	0.594	0.022	0.000	0.550	0.638
	Infection	0.536	0.023	0.127	0.491	0.580
	Response	0.487	0.025	0.573	0.438	0.536
	Organ dysfunction	0.851	0.015	0.000	0.821	0.881
	MEDS	0.758*	0.019	0.000	0.721	0.796
	APACHE II	0.764*	0.019	0.000	0.727	0.801
28-day mortality	PIRO	0.744	0.022	0.000	0.701	0.786
	Predisposition	0.555	0.025	0.029	0.507	0.603
	Infection	0.507	0.025	0.795	0.457	0.556
	Response	0.427	0.027	0.004	0.374	0.481
	Organ dysfunction	0.816	0.019	0.000	0.780	0.852
	MEDS	0.736	0.022	0.000	0.693	0.779
	APACHE II	0.742	0.022	0.000	0.700	0.784

APACHE, Acute Physiology and Chronic Health Evaluation; AUC, area under the receiver operating characteristic curve; CI, confidence interval; MEDS, Mortality in Emergency Department Sepsis; MOD, multiple organ dysfunction; PIRO, predisposition, infection, response, and organ dysfunction; SE, standard error. Compared with PIRO: **P < 0.01; *P < 0.05.

### Comparison of severity systems in patients with different outcomes

**Figure 2:**
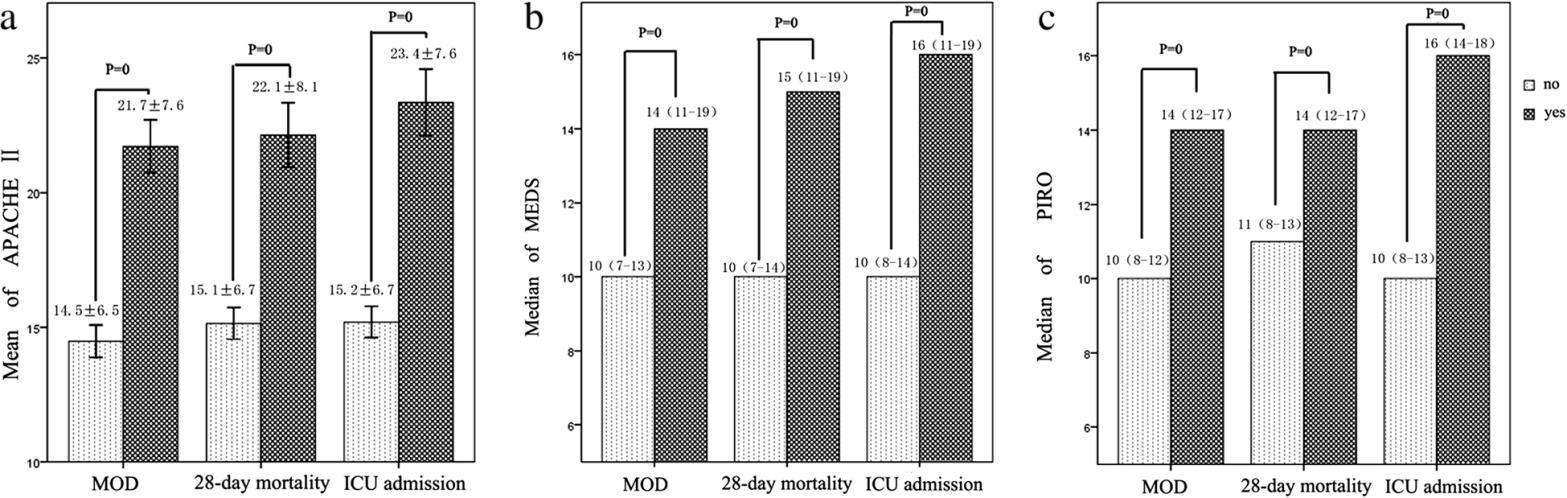
**Mean scores of severity systems between patients who did and did not meet the outcome criteria. (a)** Acute Physiology and Chronic Health Evaluation (APACHE) II score. **(b)** Mortality in Emergency Department Sepsis (MEDS) score. **(c)** Predisposition, infection, response, and organ dysfunction (PIRO) score. No, patients did not meet the outcome criteria; yes, patients met the outcome criteria. MOD, multiple organ dysfunction.

The average APACHE II, MEDS, and PIRO scores were significantly different between patients who did and did not meet the outcome criteria (*P* < 0.001). The results are shown in Figure 2. As shown in Table 5, both PIRO and APACHE II scores independently predicted ICU admission and MOD, but MEDS scores did not. All three scores were independent predictors of 28-day mortality and had similar AUC values.

**Table 5:** The independent predictors of outcomes

**Outcome variable**	**Independent predictor**	***B***	**SE**	**Wald**	***P *** **value**	**OR**	**95% CI**	
							**5%**	**95%**
ICU admission	PIRO	0.564	0.061	84.853	0.000	1.758	1.559	1.982
	MEDS	−0.020	0.033	0.393	0.531	0.980	0.919	1.044
	APACHE II	0.045	0.022	4.233	0.040	1.046	1.002	1.092
	Constant	−9.292	0.744	155.773	0.000	0.000		
MOD	PIRO	0.295	0.041	53.053	0.000	1.343	1.241	1.454
	MEDS	0.042	0.026	2.747	0.097	1.043	0.992	1.097
	APACHE II	0.065	0.017	14.260	0.000	1.067	1.032	1.104
	Constant	−5.920	0.468	160.023	0.000	0.003		
28-day mortality	PIRO	0.112	0.036	9.843	0.002	1.119	1.043	1.200
	MEDS	0.065	0.026	6.481	0.011	1.067	1.015	1.122
	APACHE II	0.075	0.017	20.207	0.000	1.078	1.043	1.114
	Constant	−4.628	.395	137.245	0.000	0.010		

APACHE, Acute Physiology and Chronic Health Evaluation; CI, confidence interval; MEDS, Mortality in Emergency Department Sepsis; MOD, multiple organ dysfunction; OR, odds ratio; PIRO, predisposition, infection, response, and organ dysfunction; SE, standard error.

The ROC curves of APACHE II and MEDS scores are shown in Figure 1, and the AUCs are presented in Table 4. The predictive ability of the PIRO score for ICU admission (AUC: 0.889) was much better than that of MEDS (AUC: 0.774) and APACHE II (AUC: 0.789) (*P* < 0.01). The PIRO score was also better for predicting the development of MOD (AUC: 0.817) than MEDS (AUC: 0.758) and APACHE II (AUC: 0.764) scores (*P* < 0.05). The AUC values of the three systems for 28-day mortality were similar.

## Discussion

It is important for the ED physician to identify high-risk septic patients who are likely to need aggressive resuscitation since significant physiologic changes from sepsis to severe sepsis and septic shock can occur rapidly in the early stages of sepsis. According to the 2012 International Guidelines for Management of Severe Sepsis and Septic Shock, resuscitation should be initiated in patients with sepsis-induced tissue hypoperfusion. In these guidelines, tissue hypoperfusion was defined as hypotension persisting after an initial fluid challenge, or a blood lactate concentration > 4 mmol/l [15]. In fact, most patients with elevated lactate are in the late stages of sepsis, and they experience high mortality despite aggressive resuscitation. Development of a more useful tool for risk stratification and prognosis in septic patients is thus still essential. For this purpose, the PIRO concept was proposed. The four components of the PIRO system cover almost all of the factors that may influence the onset, development, and outcome of sepsis. However, translating the PIRO concept into clinical practice is very difficult because of the extremely complex pathophysiologic changes of sepsis. In 2008, Moreno and colleagues developed the first PIRO score [10]. During the next 5 years, three additional clinical studies focusing on the PIRO system were published [11-13]. The enrollment populations varied from patients with clinically suspected infections to patients with severe sepsis/septic shock admitted to the ICU. The illness severity of the enrollment cohort was significantly different in every study, and the in-hospital mortality ranged from 4.3% to 48.5%. The statistical methods of screening the variables also differed between studies. The PIRO systems developed in these studies were therefore very different. Additionally, these studies focused on the prognostic value of the PIRO system, and did not assess its performance in risk stratification.

The PIRO system assessed in the present investigation was developed from a study of 2,132 patients with suspected infections, and its prognostic value for in-hospital mortality was validated in an internal cohort (*n* = 4,618) and an external cohort (*n* = 1,004). In that initial study, the AUCs of the PIRO system in predicting in-hospital mortality were 0.90 in the derivation cohort, 0.86 in the internal validation cohort, and 0.83 in the external validation cohort [12]. This PIRO system incorporated 16 variables that were easily obtained in the ED and was superior to other PIRO systems for application in the ED. As the original PIRO enrollment cohort comprised patients with suspected infection who were relatively low risk (in-hospital mortality of 4.3%), its prognostic and risk stratification performance in septic patients required further assessment.

The present study assessed the predictive ability of the four components of the PIRO system separately and found that organ dysfunction was the best predictor of outcomes. The predictive value of each of the other three components was weaker. Some variables of the PIRO system (systolic blood pressure and lactate concentration) reflected established organ dysfunction and may partly account for the superior performance of organ dysfunction in predicting ICU admission and MOD. Another important result of our study is that response negatively correlated with MOD and 28-day mortality. As the criteria of response in the original PIRO were tachycardia, tachypnea, and elevated bands, patients who did not develop these symptoms were prone to adverse outcomes in the present study. Some septic patients manifest hypothermia and leucopenia instead of fever and leukocytosis, and their illness severity is more severe with poorer outcome [16]. This result suggests that adding hyporeactive variables in the PIRO system may be necessary.

Our study revealed that APACHE II, MEDS, and PIRO scores were much higher for patients admitted to the ICU than for those who were not. In binary logistic regression analysis, the independent predictors of ICU admission were the APACHE II and PIRO scores, but not the MEDS score. This may be because more organ dysfunction variables are used in the APACHE II and PIRO scores. Most patients were admitted to the ICU because they developed organ dysfunction that needed intensive therapy such as mechanical ventilation or continuous renal replacement therapy. The MEDS score incorporates fewer variables that reflect organ function, so its predictive ability for ICU admission is weaker. The superior predictive performance of the PIRO system for ICU admission may be helpful in establishing the disposition of septic patients in the ED.

MOD signals progressive deterioration and is associated with high short-term mortality [1,3,4]. In our study, both PIRO and APACHE II scores independently predicted the development of MOD within 3 days of ED arrival, but MEDS scores did not. The predictive value of the PIRO system was superior to that of APACHE II. The PIRO system incorporates both the high-risk factors of MOD, such as age, liver disease, malignancy, and residence in a nursing home, and variables reflecting established organ dysfunction, such as systolic blood pressure and lactate; therefore, its predictive value was found to be better. The APACHE II score contains organ dysfunction variables, such as mean artery pressure, creatinine, and oxygenation, but other variables did not reflect organ function, so its predictive value was weaker compared with the PIRO score.

The PIRO system was an independent predictor of 28-mortality in the present study. Its prognostic value was similar to that of MEDS and APACHE II scores. A previous study obtained analogous results [17]. The PIRO system may be able to replace MEDS and APACHE II scores as a prognostic scoring system after validation in larger cohorts in the future.

### Limitations

Our study was limited by being a single-center study with a relatively small sample size. It is also important to recognize that the present study excluded 48 patients with terminal disease (malignant cancer with metastases, AIDS, end-stage renal or hepatic disease, chronic heart failure), which significantly influenced the short-term survival rate. Data for these patients were not recorded or analyzed. The absolute scores of MEDS, APACHE II, and PIRO may therefore be a little lower in the present study than in other studies that included terminal patients.

## Conclusions

The PIRO system is valuable in predicting 28-day mortality, ICU admission, and the development of MOD in septic patients in the ED. The PIRO score is superior to the MEDS and APACHE II scores for risk stratification, and its prognostic value is similar to MEDS and APACHE II scores. Organ dysfunction is the best predictor of ICU admission, MOD, and 28-day mortality among the four components of the PIRO system. The response component negatively correlated with MOD and 28-day mortality.

## Key messages

• Organ dysfunction is the best predictor of ICU admission, MOD, and 28-day mortality among the four components of the PIRO system.

• The predictive ability of predisposition, infection, and response components is weaker than that of organ dysfunction.

• Response negatively correlates with MOD and 28-day mortality.

• The PIRO score is superior to the APACHE II and MEDS scores for predicting ICU admission and MOD.

• The prognostic performance of the PIRO system for 28-day mortality is similar to that of the MEDS and APACHE II scores.

## Abbreviations

APACHE: Acute Physiology and Chronic Health Evaluation; AUC: area under the receiver operating characteristic curve; ED: emergency department; ICU: Intensive care unit; MEDS: Mortality in Emergency Department Sepsis; MOD: multiple organ dysfunction; PIRO: predisposition, infection, response, and organ dysfunction; ROC: receiver operating characteristic.

## Competing interest

The authors declare that they have no competing interests.

## Authors’ contributions

Y-XC conceived and designed the research, acquired, analyzed and interpreted the data, drafted the manuscript and revised it critically for important intellectual content. C-SL conceived and designed the research, analyzed and interpreted the data, gave final approval of the version to be published, and was responsible for the overall content as guarantor. All authors read and approved the final manuscript.
